# Inactivation and Inducible Oncogenic Mutation of p53 in Gene Targeted Pigs

**DOI:** 10.1371/journal.pone.0043323

**Published:** 2012-10-05

**Authors:** Simon Leuchs, Anja Saalfrank, Claudia Merkl, Tatiana Flisikowska, Marlene Edlinger, Marina Durkovic, Nousin Rezaei, Mayuko Kurome, Valeri Zakhartchenko, Barbara Kessler, Krzysztof Flisikowski, Alexander Kind, Eckhard Wolf, Angelika Schnieke

**Affiliations:** 1 Chair of Livestock Biotechnology, Technische Universität München, Freising, Germany; 2 Chair of Molecular Animal Breeding and Biotechnology, Ludwig-Maximilians-Universität München, Oberschleissheim, Germany; Virginia Commonwealth University, United States of America

## Abstract

Mutation of the tumor suppressor p53 plays a major role in human carcinogenesis. Here we describe gene-targeted porcine mesenchymal stem cells (MSCs) and live pigs carrying a latent *TP53^R167H^* mutant allele, orthologous to oncogenic human mutant *TP53^R175H^* and mouse *Trp53^R172H^*, that can be activated by Cre recombination. MSCs carrying the latent *TP53^R167H^* mutant allele were analyzed in vitro. Homozygous cells were p53 deficient, and on continued culture exhibited more rapid proliferation, anchorage independent growth, and resistance to the apoptosis-inducing chemotherapeutic drug doxorubicin, all characteristic of cellular transformation. Cre mediated recombination activated the latent *TP53^R167H^* allele as predicted, and in homozygous cells expressed mutant p53-R167H protein at a level ten-fold greater than wild-type MSCs, consistent with the elevated levels found in human cancer cells. Gene targeted MSCs were used for nuclear transfer and fifteen viable piglets were produced carrying the latent *TP53^R167H^* mutant allele in heterozygous form. These animals will allow study of p53 deficiency and expression of mutant p53-R167H to model human germline, or spontaneous somatic p53 mutation. This work represents the first inactivation and mutation of the gatekeeper tumor suppressor gene *TP53* in a non-rodent mammal.

## Introduction

Cancers are the fourth most common cause of death worldwide and predicted to increase with longer human life expectancy. Current statistics indicate that more than 40% of people alive today will be diagnosed with some form of cancer during their lifetime [1]. Years of basic research and advances in cancer genetics and medicinal chemistry have provided a wealth of knowledge that promise to revolutionize cancer detection and treatment. The challenge now is to translate these accomplishments into clinical benefit for patients in a safe and efficient manner.

The transcription factor p53 is a vital tumor suppressor that inhibits cell cycle progression following DNA damage and promotes senescence or apoptosis in response to stress. Modification of p53 function occurs in the majority of human cancers [2], and germline mutations in *TP53* are responsible for Li Fraumeni multiple cancer syndrome [3]. The location, type and frequency of *TP53* somatic mutations have been surveyed across a wide range of human cancers, 86% lie between codons 125 and 300, broadly corresponding to the DNA binding region [4]. Certain mutations exhibit a dominant negative effect and may impart gain-of-function oncogenic properties [5]. The R175H mutation is of this type. Mutant p53-R175H inhibits wild-type p53 interaction with promoter elements [6], advances angiogenesis [7], and promotes epithelial mesenchymal transition [8] important in metastasis and tumor invasion. A survey of somatic p53 mutations compiled by the International Agency for Research on Cancer reveals R175H as the most frequent missense mutation in many sporadic human cancers [9].

Many *Trp53* modifications have been generated in mice, including knockout and inducible oncogenic activation mutations [10]. Mice provide powerful tools for cancer genetics, identification of possible therapeutic targets and proof-of-principle experiments. But mice are not men, their small size and short lifespan restrict the types of study that can be carried out. There is a need for genetically defined models of cancer and cancer predisposition in other species. Larger, longer-lived animals offer the advantage that disease initiation, progression and response to treatment can be closely monitored in real time under conditions similar to human patients. Many important parameters can be followed in longitudinal studies, such as early tumor markers, the presence of circulating tumor cells, tumor progression and remission, response or failure of drug therapy, and the acquisition of drug resistance by cancer cells.

Our aim is to facilitate preclinical research and the evaluation of new diagnostic and therapeutic procedures in human oncology. Here we describe a gene-targeted pig line carrying a latent *TP53^R167H^* mutant allele orthologous to human *TP53^R175H^* that can be activated by Cre recombination to model Li Fraumeni syndrome and oncogenic mutant p53 found in sporadic human cancers.

## Results

We engineered a latent oncogenic mutant porcine *TP53* gene by insertion of a floxed transcriptional termination signal (LSL cassette) in the first intron and a point mutation encoding the oncogenic activation mutation R167H (equivalent to human R175H and mouse R172H) in exon 5. The intact allele, designated *TP53^LSLR167H^*, is predicted to lack *TP53* expression. Cre recombinase-mediated removal of the LSL cassette generates the allele designated *TP53^LR167H^*, which is predicted to express mutant p53-R167H mRNA and protein.

### Porcine *TP53* gene targeting

**Figure 1:**
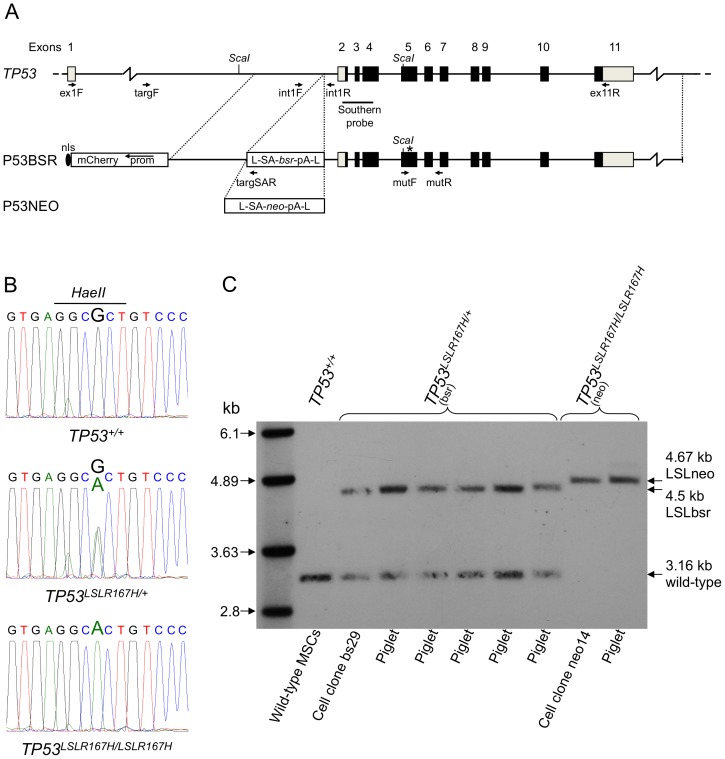
Porcine *TP53* gene targeting. A) Porcine *TP53* gene targeting scheme. Top. Porcine *TP53* gene. Exon numbers are indicated, coding and non-coding regions are marked as black and grey boxes. Below. Structure of P53BSR and P53NEO targeting vectors. The transcription termination cassette and its insertion site in intron 1 is shown, the G to A substitution mutation in exon 5 is indicated by an asterisk. Detailed descriptions of each vector are provided in materials and methods. PCR and RT-PCR primers used to identify targeted cell clones, detect mRNAs, detect presence and loss of the transcriptional termination cassette, and to amplify the mutated region in exon 5 are indicated. *ScaI* restriction sites and the hybridization probe used for Southern analysis are also shown. B) Sequence analysis across codon 167. Representative chromatograms of exon 5 region including the engineered R167H mutation showing wild-type, heterozygous and homozygous mutation sequences detected in targeted cell clones. The *HaeII* restriction enzyme site used to distinguish mutant from wild-type RT-PCR fragments is also shown. C) Southern blot analysis of cell clones and piglets. A diagnostic *ScaI* fragment was used to assess targeted integration at the *TP53* locus. Predicted sizes for *TP53* alleles are: wild-type *TP53* 3.16 kb, *TP53^LSLR167H^* (bsr) 4.5 kb, *TP53^LSLR167H^* (neo) 4.67 kb. *ScaI* digested genomic DNA from targeted cell clones and nuclear transfer derived piglets are as indicated.

Mesenchymal stem cell (MSC) isolates were prepared from the bone marrow of Landrace/Pietrain cross breed pigs, or adipose tissue of Landrace male pigs. Cell isolates capable of differentiation into osteogenic, adipogenic and chondrogenic lineages were used. MSCs were transfected separately with P53BSR or P53NEO vectors. These vectors differ only in the drug selectable marker (*bsr* or *neo*), see Figure 1A. Both employ a promoter trap strategy to enrich targeted cell clones and include the fluorescent mCherry gene as a visible counter-selectable marker to identify random integrants.

Putative gene targeted cell clones were identified by lack of mCherry fluorescence and PCR amplification of a 3.3 kb DNA fragment from the LSL cassette across the shorter arm of homology to a point outside the targeting vector, see Figure 1A. One of eight P53NEO transfectants and seven of 78 P53BSR transfectants were identified as targeted, an overall targeting efficiency of 9.3%. Representative screening results are shown in Figure S1. The presence of the G to A mutation (encoding R167H) in exon 5 was tested by PCR amplification and sequence analysis across the mutation site. Six cell clones with both G and A nucleotides at the mutation site, indicating heterozygosity, were identified. The P53NEO targeted cell clone (neo14) showed only the A nucleotide, see Figure 1B, indicating either homo- or hemizygosity. Subsequent analysis confirmed that this clone was *TP53^LSLR167H/LSLR167H^* homozygous, as described later. Sequence analysis also indicated loss of 223 bp intron sequence adjacent to the LSL cassette in this clone in both alleles.

### *TP53* gene targeted pigs

One nuclear transfer experiment was carried out with the single *TP53^LSLR167H/LSLR167H^* bone marrow derived cell clone neo14. Embryos were transferred to one recipient and one pregnancy established. Two fully developed male piglets with normal birth weights (1.2 and 1.7 kg) were born, however these exhibited macroglossia causing asphyxia and death at birth. This abnormality has previously been noted after nuclear transfer in pigs [11] and other mammalian species [12], and is understood to be a consequence of defective epigenetic reprogramming. It is almost certainly related to the cell type used and not the *TP53* mutation, because animals derived from the same bone marrow MSC preparation for another targeting project showed a similar defect. No further nuclear transfer experiments were carried out with these cells.

Two sets of nuclear transfer experiments were carried out with the heterozygous *TP53^LSLR167H/+^* adipose derived cell clones bs29 and bs33. Clones bs29 and bs33 were chosen because the original colonies were well separated and unequivocally free of mCherry fluorescent cells. They were pooled for nuclear transfer to increase the chance of including a competent donor. Reconstructed embryos were transferred to six recipients, four pregnancies established and a total of 15 piglets born, all male. In contrast to nuclear transfer with bone marrow cells, all 15 piglets were free of any apparent abnormality and remain so at the time of writing.

Piglets were first analyzed by PCR to detect targeted insertion of the LSL cassette and presence of the R167H mutation. Both animals from clone neo14 were confirmed as *TP53^LSLR167H/LSLR167H^* and all 15 animals from clones bs29/bs33 as *TP53^LSLR167H/+^*. Figure 1C shows Southern blot analysis of cell clones neo14 and bs29 and representative nuclear transfer derived piglets. A diagnostic *ScaI* fragment was used to indicate insertion of the LSL cassette (see Figure 1A). Fragments indicative of wild-type and targeted *TP53* alleles were detected in *TP53^LSLR167H/+^* animals, while neo14 derived *TP53^LSLR167H/LSLR167H^* piglets did not show the wild-type fragment. The internal probe used (Figure 1A) would also have revealed any random integrations of the targeting vector, none were detected.

A series of experiments were carried out to investigate the function and phenotype of the targeted *TP53* allele in vitro, in preparation for in vivo characterization of the phenotype in whole animals.

### Activation of targeted *TP53* allele by Cre transduction

**Figure 2:**
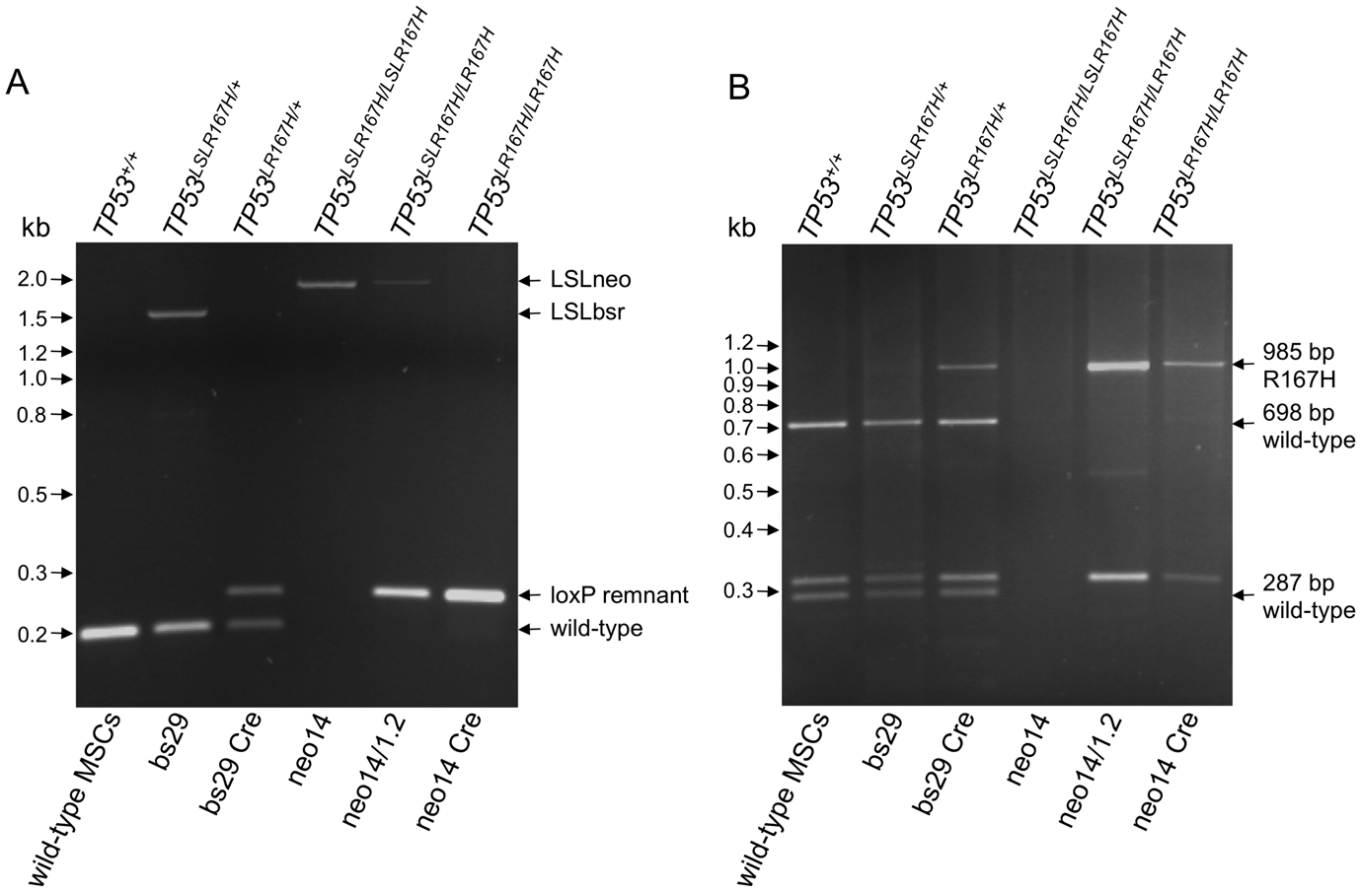
Blockade and release of the *TP53^LSLR167H^* allele. A) Cre recombination removes the LSL cassette from the *TP53^LSLR167H^* allele. Gel analysis of PCR amplification products across the site of LSL insertion in porcine TP53 intron 1 in: wild-type MSCs, *TP53^LSLR167H/+^* cell clone bs29 before and after Cre transduction; the *TP53^LSLR167H/LSLR167H^* cell clone neo14, the partially Cre-recombined cell subclone neo14/1.2 (*TP53^LSLR167H/LR167H^*); and cell clone neo14 after Cre transduction (*TP53^LR167H/LR167H^*) as indicated. Predicted fragment sizes are: wild-type *TP53* 198 bp, non-excised LSL (*bsr*) 1.536 kb, non-excised LSL (*neo*) 1.929 kb. Cre-mediated LSL excision was predicted to leave a 34 bp loxP site and a 22 bp vector remnant that lies outside the floxed region; thus a 254 bp amplified fragment. B) Cre recombination activates mutant p53-R167H mRNA expression. Gel analysis of RT-PCR amplification products of mRNAs (exon 1 to exon 11) expressed from wild-type and activated mutant *TP53* alleles. RT-PCR products are distinguished by digestion with *HaeII*. The G to A mutation in codon 167 removes a *HaeII* site present in wild-type p53, see Figure 1B. Lanes show wild-type MSCs, *TP53^LSLR167H/+^* cell clone bs29 before and after Cre transduction; *TP53^LSLR167H/LSLR167H^* cell clone neo14, the partially Cre-recombined cell subclone neo14/1.2 (*TP53^LSLR167H/LR167H^*); and cell clone neo14 after Cre transduction (*TP53^LR167H/LR167H^*) as indicated. Predicted sizes for *HaeII* digested amplified RT-PCR fragments are: wild type *TP53* 698 bp, 310 bp, 287 bp and 18 bp; Cre-recombined *TP53LR^167H^* allele 985 bp, 310 bp and 18 bp.

The floxed transcription termination cassette in intron 1 was predicted to block expression of the *TP53^LSLR167H^* allele, and the block released by Cre-mediated excision. We examined the effects of Cre protein transduction into targeted cell clones bs29 and neo14. As shown in Figure 2A, non-targeted MSCs amplified only the wild-type *TP53* fragment. Heterozygous *TP53^LSLR167H/+^* cell clone bs29 amplified wild-type and non-excised LSL (*bsr*) fragments before Cre transduction, and wild-type and excised loxP remnant fragments after transduction. *TP53^LSLR167H/LSLR167H^* cell clone neo14 amplified only the non-excised LSL (*neo*) fragment before Cre transduction, and only the excised loxP remnant fragment after transduction.

Cre recombination was also used to confirm that clone neo14 was *TP53^LSLR167H/LSLR167H^* homozygous, rather than *TP53^LSLR167H/−^* hemizygous. Neo14 cells were subjected to Cre transduction, individual G418 resistant subclones selected and isolated and their TP53 genotype analyzed. Two subclones showed both a non-excised LSL (*neo*) and a loxP remnant fragment, indicating that the parental clone neo14 possessed two gene-targeted *TP53* alleles. Figure 2A shows *TP53^LSLR167H/LR167H^* subclone neo14/1.2.

The G to A substitution in codon 167 results in loss of a *HaeII* restriction enzyme recognition site, see Figure 1B. We used RT-PCR amplification of a 1.313 kb RT-PCR product from exon 1 to exon 11 to detect p53 mRNA expression and *HaeII* digestion to distinguish mutant and wild-type p53. Figure 2B shows *HaeII* RT-PCR analysis of *TP53^LSLR167H/+^* cell clone bs29 and *TP53^LSLR167H/LSLR167H^* cell clone neo14 before and after Cre recombination, and also the partially recombined *TP53^LSLR167H/LR167H^* subclone neo14/1.2. The characteristic *HaeII* fragments are indicated. Results clearly indicate that the *TP53^LSLR167H^* allele lacks p53 mRNA expression, and is thus a null allele. Cre mediated excision of the LSL cassette activates mutant p53-R167H mRNA expression. This demonstrates tight blockade and release of the targeted allele.

### Elevated levels of mutant p53-R167H protein in porcine MSCs

**Figure 3:**
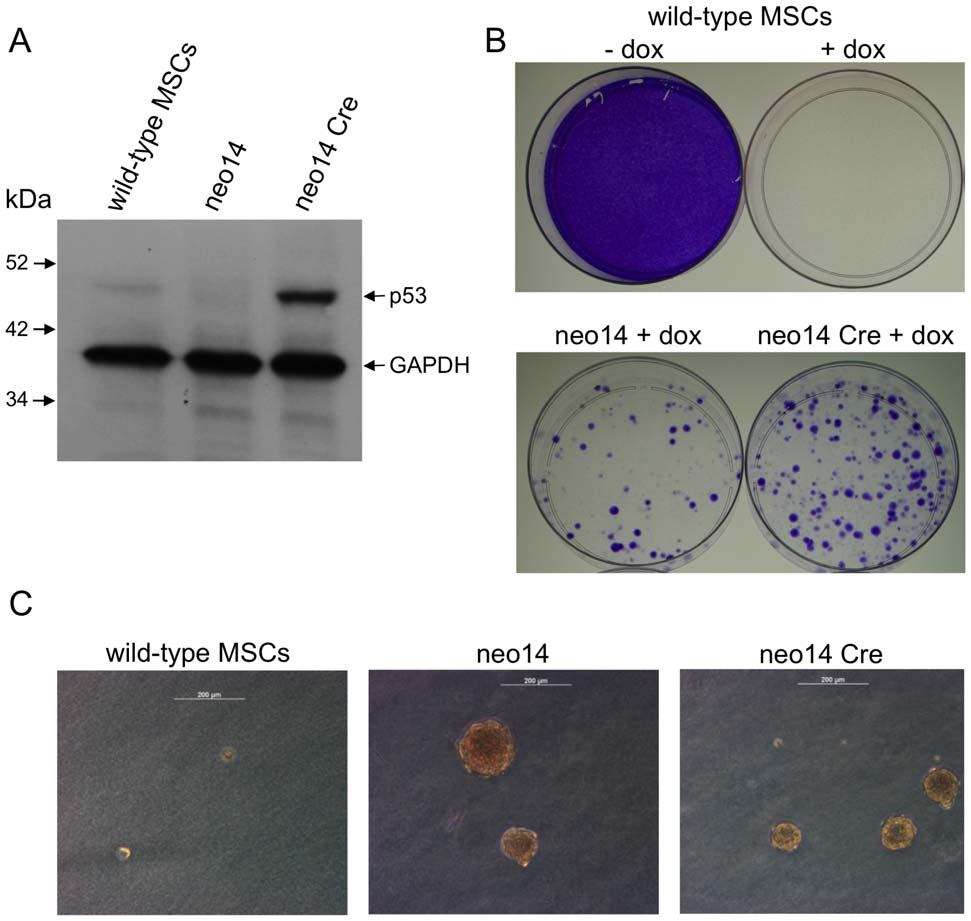
Porcine p53 deficiency and mutant p53-R167H expression cause phenotypic changes in vitro. A) Cre recombination activates abundant mutant p53-R167H protein expression. The Western blot shows detection of p53 and GAPDH in wild-type MSCS and the cell clone neo14 before (*TP53^LSLR167H/LSLR167H^*) and after Cre transduction (*TP53^LR167H/LR167H^*) as indicated. Protein size markers are shown. B) p53 deficiency confers resistance to doxorubicin, this is not reversed by mutant p53-R167H expression. Top, wild-type porcine MSCs grown for 14 days untreated and after treatment with doxorubicin, as indicated. Bottom, cell clone neo14 before (*TP53^LSLR167H/LSLR167H^*) and after Cre transduction (*TP53^LR167H/LR167H^*) grown for 14 days after treatment with doxorubicin. Cells are stained with crystal violet. C) Anchorage independent growth of p53 deficient and mutant p53-R167H expressing cells. Representative colonies after 4 weeks growth in soft agar. Wild-type MSCs, cell clone neo14 before (*TP53^LSLR167H/LSLR167H^*) and after Cre transduction (*TP53^LR167H/LR167H^*) as indicated. Scale bar is 200 µm.

Wild-type p53 protein is maintained at low level in normal cells, mainly by MDM2-mediated ubiquitination and degradation [13]. Some mutant p53 proteins, such as human R175H, lack the ability to transactivate MDM2 and thus accumulate in cancer cells [14]. We assessed whether the porcine R167H mutation has a similar effect. Figure 3A shows Western blot analysis of wild-type MSCs and cell clone neo14 before and after Cre transduction. p53 protein expression is evident in wild-type MSCs, but absent in neo14. Following Cre transduction, neo14 cells express abundant p53 protein. Gel density analysis of three Western blots showed approximately ten-fold more mutant p53-R167H in induced neo14 cells than wild-type p53 in untargeted MSCs; data were normalized to the GAPDH signal. The ∼46 kDa size we observed for porcine p53 is consistent with that previously reported [15]. These data indicate that, like human R175H and mouse R172H, the porcine R167H mutation causes p53 accumulation.

### p53 deficiency results in transformation of porcine MSCs in vitro

Inactivation of p53 in mouse MSCs results in cell transformation [16]. Cell clone neo14 provided an opportunity to investigate p53 deficiency in porcine MSCs. Most porcine primary cells can only be maintained for a short time after genetic manipulation and single cell cloning because, like most untransformed primary cells, they undergo replicative senescence. This was not the case with the *TP53^LSLR167H/LSLR167H^* cell clone neo14. After a short time in culture (2 weeks after single cell cloning), neo14 cells exhibited a noticeable change in growth characteristics. The rate of proliferation increased compared to wild-type MSCs and *TP53^LSLR167H/+^* cell clones, with the time required for cell doubling decreasing from ∼72 h to ∼24 h. Cell morphology also changed from fibroblast-like to a smaller, round shape. Unlike the parental MSCs, this cell clone could sustain multiple rounds of subcloning, and has been passaged at 1∶10 dilution more than 60 times with no evidence of replicative senescence.

The ability of individual cells to form colonies in soft agar was investigated as a simple, standard test of cellular transformation. Wild-type MSCs, *TP53^LSLR167H/LSLR167H^* neo14 p53 deficient cells, and Cre transduced *TP53^LR167H/LR167H^* neo14 cells were compared. Four weeks after plating 1×10^3^ cells no colonies greater than 50 µm diameter were obtained from wild-type MSCs. Neo14 produced an average of 70 (+/−7) and Cre-transduced neo14 cells an average of 100 (+/−22) colonies, based on three replicate experiments. Figure 3C illustrates typical colony appearance.

### p53 deficiency confers chemoresistance in vitro

p53 deficiency or oncogenic p53 mutations are known to confer increased resistance to the chemotherapeutic drugs such as cisplatin and doxorubicin in mouse and human cells [17], [18]. We used a doxorubicin assay to investigate p53-mediated apoptosis. As Figure 3B shows, wild-type porcine MSCs were completely killed by doxorubicin. The p53 deficient cell clone neo14 showed resistance, and this was not affected by inducing mutant p53-R167H expression, as shown by the doxorubicin resistance of Cre-transduced neo14 cells.

These in vitro data show that the *TP53^LSLR167H^* allele is tightly repressed and can readily be activated by Cre recombination. p53 deficiency results in phenotypic changes in porcine primary cells consistent with oncogenic transformation. Expression of mutant p53-R167H results in overabundance of the protein and no reversal of the phenotypic changes, indicating that mutant p53-R167H lacks normal p53 function.

## Discussion

With a few exceptions, cancer mortality statistics have shown little improvement over the past twenty years and there is a clear need to translate novel diagnostic and therapeutic strategies into the clinic. The need to move beyond the current heavy reliance on rodent models for drug and medical device development is increasingly recognized by preclinical researchers and pharmaceutical companies. The pig is very suitable for translational research [19], [20] because of its physiological and pharmacological similarities to humans. Here we describe an important new resource for preclinical oncology, pigs with a latent oncogenic mutant p53-R167H (orthologous to human R175H and mouse R172H) that can be activated by Cre recombinase, or in its unrecombined form be used as a *TP53* null allele. *TP53* is mutated or silenced in the majority of human cancers and mutations in *TP53* play a central role in evading growth suppression in tumor progression and metastasis. In some cases, for example in Li-Fraumeni syndrome, inactivation or expression of oncogenic p53 can be the initiating factor of tumorigenesis [3]. The p53 pathway is therefore an attractive candidate for anti-cancer therapy [21].

Our data show that *TP53* targeting in MSCs was efficient. Using promoter trap vectors, 9.3% of primary cell clones analyzed showed correct gene targeting, 86% of clones found to contain the LSL cassette had also incorporated the R167H mutation. Moreover one clone showed the targeted LSL insertion and R167H mutation in both alleles. While the bone marrow MSC isolate used to derive this clone was not fully competent for the production of viable piglets, this cell clone provided valuable data in vitro, demonstrating that p53 inactivation and mutation in porcine primary MSCs results in a transformed phenotype and resistance to chemotherapeutic drug-induced apoptosis.

This suggests that p53 inactivation may also cause tumor initiation in vivo in homozygous knockout pigs. We expect such animals to be viable, as shown for mice [22] and rats [23]. Production of nuclear transfer pigs from heterozygous targeted cells (*TP53^LSLR167H/+^*) derived from adipose MSCs was very efficient, with four of six recipient sows becoming pregnant and 15 healthy piglets obtained.

As yet the *TP53^LSLR167H/+^* piglets show no signs of abnormalities, but are being continuously monitored because *TP53* hemizygous humans, mice and rats show increased rates of tumorigenesis [23]–[25]. Significant phenotype differences have been observed between species [23] [26], and the new porcine p53 animal models will enable interspecies comparison.

Conditional gene activation is a well established part of cancer modeling in mice [27], [28], but has not yet been extended to large animals. We have demonstrated Cre activation of the latent *TP53^LSLR167H^* allele and provided the first evidence that porcine R167H mutation is functionally equivalent to orthologous mutations in human and mouse cancer cells. We are thus confident that in vivo activation of the R167H mutation will result in a similar dominant negative effect in the whole animal. Combining the inducible *TP53* mutant allele with Cre expression systems will provide temporal and tissue specific control over the activation of mutant oncogenic p53 in vivo to mimic somatic mutations responsible for human tumor formation.

Predictive preclinical animal trials are vital for cancer research and the efficient transfer of new diagnostic and treatment procedures into the clinic. The need for animal models at human scale to bridge the gap between rodent models and human patients is increasingly recognized. We are thus engaged in an integrated program of targeted mutation of key tumor suppressor and proto-oncogenes to provide a series of genetically-defined porcine models of human cancers and cancer predispositions. We recently reported pigs carrying gene-targeted mutations in the *adenomatous polyposis coli* (*APC*) gene to model predisposition to colorectal cancer [29]. The *TP53* mutant pig line we describe here is a central component in this campaign. As with rodents, precisely engineered mutations can be brought into combination to model a variety of human cancers. We are confident that the pigs described here will provide powerful new resources for preclinical oncology and basic cancer research.

## Materials and Methods

Animal experiments were approved by the Government of Upper Bavaria (permit number 55.2-1-54-2532-34-09) and performed according to the German Animal Welfare Act and European Union Normative for Care and Use of Experimental Animals. Chemicals were obtained from Sigma-Aldrich Chemie GmbH, cell culture media and supplements from PAA Laboratories GmbH unless otherwise specified.

### Porcine *TP53* gene targeting constructs

A porcine BAC clone (GenBank: AC127472.4) was used to generate two gene-targeting vectors P53BSR and P53NEO. These differed only in the drug selectable marker gene, see Figure 1A. Each comprised: a 196 bp dual SV40 nuclear localization sequence to facilitate nuclear entry of vector DNA (Dean et al. 1999); a 2.920 kb CAGGS promoter-mCherry cassette (in reverse orientation) as a fluorescent counter-selectable marker; a 1.213 kb 5′ short arm of homology corresponding to a region of *TP53* intron 1 from a point 1.447 kb 5′ of exon 2 to a *PmlI* restriction enzyme site 234 bp 5′ of exon 2; a floxed transcriptional termination cassette (LSL); and a 10.96 kb region extending from the *PmlI* site in intron 1 to a point 5.957 kb 3′ of exon 11 that includes a G to A substitution in exon 5 changing arginine to histidine in codon 167 (R167H). The 1.3 kb LSL cassette of vector P53BSR comprised: a loxP site; adenoviral splice acceptor; promoterless blasticidin resistance gene (*bsr*); three poly-adenylation signals derived from SV40, bovine growth hormone and cytomegalovirus; and a second loxP site. The 1.7 kb LSL cassette of vector P53NEO was the same, but contained a promoterless neomycin resistance gene (*neo*).

### Generation of gene targeted porcine mesenchymal stem cell (MSC) clones

MSCs were isolated by standard methods from leg bones or subcutaneous fat of 6 to 7 month old male Landrace/Pietrain or Landrace pigs. MSCs were cultured in advanced Dulbecco's modified Eagle medium (DMEM) (Gibco), 2 mM GlutaMAX, 1× non-essential amino acids, 10% fetal calf serum (FCS gold, lot no. A15109-2859), 5 ng/ml FGF-2 (PromoKine) at 37°C, 5% CO_2_ and passaged using Accutase. Samples of 1×10^6^ MSCs were electroporated with 10 µg linearized targeting vector DNA and selected with either 8 µg/ml blasticidin (Invivogen), or 600 µg/ml G418. Colonies were examined by fluorescence microscopy to visually identify and exclude those that expressed the mCherry counter-selectable marker. Individual stable transfected cell clones were isolated, samples of each clone cryopreserved at an early stage and replicate samples cultured further for DNA, RNA and protein analyses.

### PCR and RT-PCR analysis of targeted MSC clones

Cell clones carrying the LSL cassette were identified using primer targF (5′ CCAGGGAGTCCATCTAAAAGTG 3′), which hybridizes to a point in intron 1 outside the targeting vector, and primer targSAR (5′ GAAAGACCGCGAAGAGTTTG 3′), which hybridizes to the LSL splice acceptor. PCR was carried out with GoTaq polymerase (Promega). Thermal cycling parameters were: 5 min, 95°C; then 40 cycles of: 30 sec, 95°C; 30 sec, 59°C; 3:30 min, 72°C; followed by 5 min, 72°C. The diagnostic amplified product was 3.308 kb.

The wild-type *TP53* allele was amplified using primer targF (sequence above) and primer int1R (5′ TTCCACCAGTGAATCCACAA 3′). Thermal cycling parameters were as above. The diagnostic amplified product was 3.16 kb.

The presence of the G to A substitution in exon 5 was detected by PCR amplification across the mutation site using primers mutF (5′ GTACTCCCCTGCCCTCAATA 3′) and mutR (5′ GGGGTAACCCATCTGCTCTA 3′) followed by DNA sequence analysis. PCR was carried out with GoTaq polymerase. Thermal cycling parameters were: 5 min, 95°C; then 35 cycles of: 30 sec, 95°C; 30 sec, 59°C; 30 sec, 72°C; followed by 5 min, 72°C.

Cre-mediated excision of the LSL cassette was detected by PCR across the LSL insertion site using primers int1F (5′ TGAGGAATTTGTATGCCAAGG 3′) and int1R (sequence above) with GoTaq polymerase. Thermal cycling parameters were: 5 min, 95°C; then 30 cycles of: 30 sec, 95°C; 30 sec, 58°C; 1:30 min, 72°C; followed by 5 min, 72°C.

mRNAs expressed from wild type *TP53* and the Cre-recombined *TP53^LR167H^* allele were detected by RT-PCR amplification of a 1.313 kb fragment from exon 1 to exon 11 by two step RT-PCR with Superscript III RT according to the manufacturer's instructions, using primers ex1F (5′ GCAGGTAGCTGCTGGTCTC 3′ ) and ex11R (5′ AGGGACTTCAAAAGGGGATG 3′) with GoTaq polymerase. Thermal cycling parameters were: 3 min, 95°C; then 35 cycles of: 30 sec, 95°C; 30 sec, 58°C; 1:30 min, 72°C; followed by 3 min, 72°C. Wild-type p53 and mutant p53-R167H RT-PCR products containing the G to A substitution mutation were distinguished by *HaeII* (NEB) digestion.

### Somatic cell nuclear transfer

Nuclear transfer and embryo transfer were performed as previously described [30]. Between 80–120 reconstructed embryos were transferred to each recipient sow.

### Southern blot analysis

*ScaI* (NEB) digested genomic DNA from cell clones or piglet ear tip samples was electrophoresed, membrane bound, hybridized and probe detected with anti-digoxigenin antibody Fab fragments conjugated with alkaline phosphatase (Roche) by standard methods. The 517 bp *TP53* hybridization probe was generated using primers probeF (5′ GCAATGGAGGAGTCGCAGT 3′) and probeR (5′ CTGCCAGGGTAGGTCTTCTG 3′) incorporating alkali labile digoxigenin-11-dUTP (Roche). Thermal cycling parameters were: 2 min, 95°C; then 35 cycles of: 30 sec, 95°C; 30 sec, 62°C; 40 sec, 72°C; followed by 8 min, 72°C. The location of the hybridization probe is indicated in Figure 1A.

### Cre transduction

Cre protein was produced in vitro with the vector pTriEx-HTNC (Addgene plasmid 13763) according to the method described by Peitz et al. [31] and Münst et al. [32]. Samples of 4×10^4^ cells in a 12 well dish were cultured with 5 µM purified Cre recombinase in culture medium with reduced (0.5%) serum for 8 hours. Medium was replaced with standard medium and culture continued.

### Western blotting

Cultured cells were dissociated mechanically in 50 mM HEPES, 150 mM NaCl, 1 mM EDTA, 0.5% IGEPAL CA-630, 10% glycerol, pH 7.9, containing 1× phosphatase inhibitor and 1× complete mini protease inhibitor (Roche). Protein was quantified using advanced protein assay reagent (Cytoskeleton Inc.). 40 µg total protein was loaded in each lane and separated by 15% SDS-PAGE. Blotting and detection of porcine p53 and GAPDH was carried out as previously described [33]. Band densities were quantified using the image analysis tool ImageJ (http://rsb.info.nih.gov/ij/).

### Soft agar growth assay

To investigate anchorage-independent growth, samples of 1×10^3^ cells were seeded on 6 well dishes into a layer of 0.4% Noble agar in culture medium (DMEM, 10% fetal calf serum, 2 mM GlutaMAX, 1× non-essential amino acids, 44 mM NaHCO3, 1% Pen/Strep) overlying 0.6% bottom agar in culture medium. Standard culture medium was added to the cells and culture proceeded for 4 weeks. Colonies greater than 50 µm were counted.

### Doxorubicin assay

For each assay, 5×10^5^ cells were plated on a 10 cm culture dish and treated with 1 µg/ml doxorubicin-hydrochloride for 24 h. Cells were then cultured under standard conditions with regular exchange of medium. After 14 days, colony growth was visualized by staining with 0.5% crystal violet in 20% methanol.

## Supporting Information

Figure S1 **Representative primary screening of transfected cell clones.** The upper panel shows PCR detection of a diagnostic 3.31 kb fragment indicating targeted insertion of the LSL cassette in a series of P53BSR transfected cell clones including bs29 and bs33 (underlined) that were later used for nuclear transfer. A plasmid that partially mimicked the structure of the targeted *TP53* locus was diluted in porcine genomic DNA and used as a positive control, as indicated (+ve). The lower panel shows amplification of a 3.16 kb fragment from wild-type TP53 from the same cell clones. Size markers are indicated. The ethidium bromide fluorogram is shown in negative for clarity.
(TIF)
