# Supplementary figures and images for: Inactivation and Inducible Oncogenic Mutation of p53 in Gene Targeted Pigs
Source: PLoS One. 2012 Oct 5;7(10):e43323. doi: 10.1371/journal.pone.0043323 (PMC3465291; doi:10.1371/journal.pone.0043323)

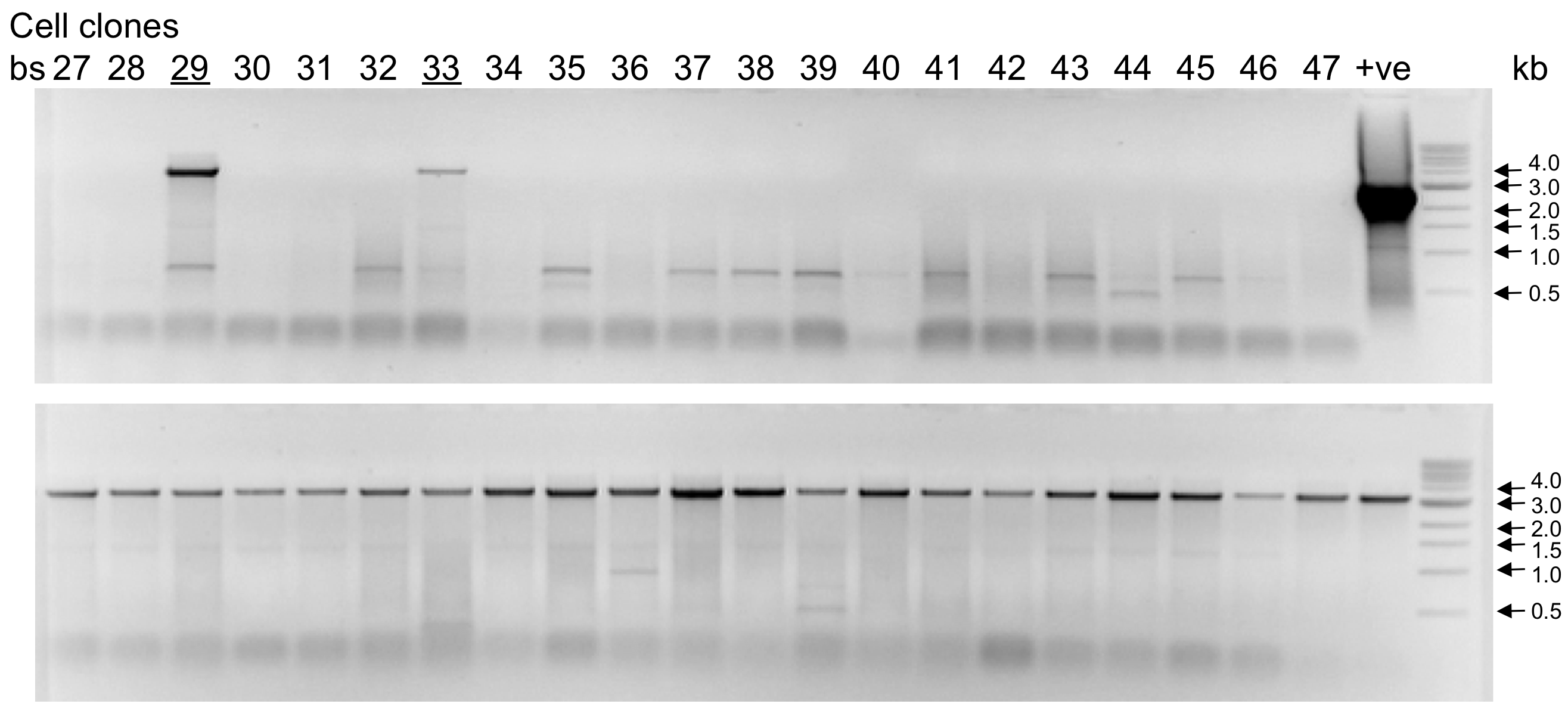

Supplement: Figure S1 — Representative primary screening of transfected cell clones. The upper panel shows PCR detection of a diagnostic 3.31 kb fragment indicating targeted insertion of the LSL cassette in a series of P53BSR transfected cell clones including bs29 and bs33 (underlined) that were later used for nuclear transfer. A plasmid that partially mimicked the structure of the targeted TP53 locus was diluted in porcine genomic DNA and used as a positive control, as indicated (+ve). The lower panel shows amplification of a 3.16 kb fragment from wild-type TP53 from the same cell clones. Size markers are indicated. The ethidium bromide fluorogram is shown in negative for clarity. (TIF) [file pone.0043323.s001.tif]
